# Genome-Wide and Transcriptome-Wide Association Studies on Northern New England and Ohio Amyotrophic Lateral Sclerosis Cohorts

**DOI:** 10.1212/NXG.0000000000200188

**Published:** 2024-09-06

**Authors:** Siting Li, Jiang Gui, Michael N. Passarelli, Angeline S. Andrew, Kathleen M. Sullivan, Kevin A. Cornell, Bryan J. Traynor, Ali Stark, Ruth Chia, Rebecca M. Kuenzler, Erik P. Pioro, Walter G. Bradley, Elijah W. Stommel

**Affiliations:** From the Departments of Biomedical Data Science (S.L., J.G.), Epidemiology (S.L., M.N.P.), and Neurology, Geisel School of Medicine at Dartmouth (E.W.S.), Dartmouth College, Hanover; Dartmouth Health (A.S.A., K.M.S., K.A.C., E.W.S.), Lebanon, NH; Neuromuscular Diseases Research Section (B.J.T., A.S., R.C.), National Institute on Aging; National Institute of Neurological Disorders and Stroke (B.J.T.), National Institutes of Health, Bethesda; RNA Therapeutics Laboratory (B.J.T.), National Center for Advancing Translational Sciences, National Institutes of Health, Rockville, MD; Cleveland Clinic (R.M.K.), OH; Department of Medicine (E.P.P.), University of British Columbia, Vancouver, BC, Canada; and University of Miami Miller School of Medicine (W.G.B.), FL.

## Abstract

**Background and Objectives:**

Amyotrophic lateral sclerosis (ALS) is an age-associated, fatal neurodegenerative disorder causing progressive paralysis and respiratory failure. The genetic architecture of ALS is still largely unknown.

**Methods:**

We performed a genome-wide association study (GWAS) and transcriptome-wide association study (TWAS) to understand genetic risk factors for ALS using a population-based case-control study of 435 ALS cases and 279 controls from Northern New England and Ohio. Single nucleotide polymorphism (SNP) genotyping was conducted using the Illumina NeuroChip array. Odds ratios were estimated using covariate-adjusted logistic regression. We also performed a genome-wide SNP-smoking interaction screening. TWAS analyses used PrediXcan to estimate associations between predicted gene expression levels across 15 tissues (13 brain tissues, skeletal muscle, and whole blood) and ALS risk.

**Results:**

GWAS analyses identified the p.A382T missense variant (rs367543041, *p* = 3.95E-6) in the *TARDBP* gene, which has previously been reported in association with increased ALS risk and was found to share a close affinity with the Sardinian haplotype. Both GWAS and TWAS analyses suggested that *ZNF235* is associated with decreased ALS risk.

**Discussion:**

Our results support the need for future evaluation to clarify the role of these potential genetic risk factors for ALS and to understand genetic susceptibility to environmental risk factors.

## Introduction

Amyotrophic lateral sclerosis (ALS) is an age-associated, fatal neurodegenerative disorder causing progressive paralysis and respiratory failure.^1,2^ With approximately 400,000 individuals worldwide estimated to be afflicted with ALS by 2040,^2^ ALS is the third most prevalent neurodegenerative disorder after Alzheimer disease (AD) and Parkinson disease (PD).^3,4^ Family history of ALS is identifiable in approximately 10% of ALS cases, with the remaining 90% being of sporadic origin.^2,5^ In addition, a recent study found that ALS has an elevated prevalence in Northern New England compared with other regions in the United States.^6^ Therefore, it is important to understand genetic and environmental risk factors specific to this region.

Since 1993, researchers have made substantial progress in unraveling genetic mechanisms involved in ALS development and progression, leading to the identification of over 2 dozen genes associated with the disorder.^2,7^ Recent progress in genotyping and sequencing technologies has improved our understanding of ALS pathology.^3^ Multiple studies have revealed overlapping susceptibility variants of ALS with other neurodegenerative diseases, including frontotemporal dementia (FTD), AD, and PD.^1,5,8^ Exploring genetic risk factors for ALS can help uncover etiopathogenic mechanisms across the spectrum of neurodegenerative diseases and potentially unveil fundamental processes involved in neuronal degeneration.^1^

Genome-wide association studies (GWAS) have played an essential role in identifying common ALS-susceptibility variants, but many of these variants have small effect sizes and are located within noncoding genomic regions.^8-10^ Transcriptome-wide association studies (TWAS) can clarify the association between genetically regulated gene expression and ALS risk and potentially identify novel genes related to ALS risk while reducing the multiple testing burden.^11^ Previous TWAS have successfully identified several ALS-associated genes expressed in various brain-related tissues and blood.^11,12^

Despite multiple studies indicating that ALS has a moderately high heritability (40%–60%),^12,13^ previously identified loci only account for a small proportion of the overall genetic predisposition to ALS.^3,5,7^ Smoking is a known risk factor for ALS.^14^ It may interact with genetic factors to influence the risk of developing ALS. For instance, smoking has been shown to induce oxidative stress, which is associated with higher ALS risk.^14^ Therefore, smoking may interact with the ALS risk gene *SOD1*, which plays a critical role in regulating oxidative stress.^15^ There are very few previous studies examining genome-wide smoking-gene interactions associated with ALS risk and our research aims to bridge this gap. This study, seeking more insight into ALS's genetic architecture, integrates GWAS and TWAS methods to detect the genetic risk factors and assesses gene-smoking interactions using sporadic ALS cases and controls based in Northern New England and Ohio that have been collected in part from previous studies.

## Methods

### Study Population

The enrollment procedure for ALS cases and controls is outlined by Andrew et al.^16,17^ In summary, we recruited ALS cases and controls from Northern New England and Ohio, with their signed consent to provide blood or saliva samples, demographic and clinical information, and complete the environmental questionnaire. Cases were newly diagnosed patients with ALS from medical centers in these regions. Controls consist of both population controls and clinic controls. Population controls were recruited randomly by mail using the US Postal Service Delivery Sequence File (USPS DSF^2^). Clinic controls were patients diagnosed with non-neurodegenerative diseases. All participants were at least aged 18 years.

Between 2020 and 2023, the Laboratory of Neurogenetics at the National Institute on Aging genotyped DNA on 435 ALS cases and 279 controls from Northern New England and Ohio participants. Genotyping was conducted using the Illumina NeuroChip according to the manufacturer's instructions, a platform designed to target curated variants in neurologic diseases.^18^ We measured genotypes for 487,374 single nucleotide polymorphisms (SNPs) from the arrays prior to quality control filtering, including 305,670 SNPs from a GWAS backbone and 179,467 custom SNPs selected throughout the genome.

### Quality Control and Genotype Imputation

We used PLINK^19,20^ software to perform standard quality control procedures for genotype data, and we implemented the following steps outlined by Chia et al.^21^ Briefly, we excluded samples with over 5% missing genotypes based on the sample call rate and removed samples with heterozygosity values beyond a threshold (F > 0.15 or F < −0.15). We removed non-European individuals from the principal component analysis because of the low numbers in the New Hampshire population, using the HapMap 3 Genome Reference Panel^22^ as the reference for ancestral information. Given that most instances of ALS are sporadic, we excluded all familial ALS cases; however, we cannot entirely exclude the rare occurrence of a monogenic gene variant because when this study was performed, sporadic cases were not undergoing clinical genetic testing. In addition, we removed variants (1) containing over 5% missing genotypes, (2) with less than 5% minor allele frequency, (3) showing deviation from Hardy-Weinberg equilibrium (*p* < 1.0E-3), and (4) with a *p*-value below 1E-4 in the case/control nonrandom missingness test.

After quality control, 613 individuals, including 378 sporadic ALS cases and 235 controls, were included in analyses, and 242,090 SNPs were available for imputation. We conducted genotype imputation by Michigan Imputation Server^23^ in GRCh37/hg19, using the European population data of the 1000 Genomes Project^24,25^ (phase 3, version 5, available at reference 26) as the imputation reference. Only SNPs with an imputation accuracy R^2^ ≥ 0.3 were included in analyses. The quantile-quantile (QQ) plots show no genomic inflation after quality control (eFigure 1).

### Genome-Wide Association Analysis

For the GWAS analysis, we performed covariate-adjusted logistic regression using PLINK, adjusting for sex, age at symptom onset, and the first 10 principal components of genetic ancestry. The Manhattan plot was generated using the “CMplot”^27^ package in R version 4.0.2. We validated the significant SNP identified in previous GWAS results using publicly available data with a larger sample size, conducting logistic regression and adjusting for the same covariates. For this validation analysis, genotype data were obtained from 10,067 ALS cases and 2,251 controls from the database of Genotypes and Phenotypes (dbGaP)^28^ with the study accession number phs000101.v5.p1. To balance the case-control ratio, we included an additional 11,887 controls from 2 other dbGaP data sets (phs000187 and phs000428). After quality control, 22,419 individuals and 335,021 variants were available for imputation in the validation study. The genotype imputation was also conducted on this validation data using the Michigan Imputation Server. A threshold *p*-value of 5E-8 was set for genome-wide significance after Bonferroni correction for multiple testing in the GWAS.

In addition to examining the main effect of SNPs, we also evaluated SNP-smoking interactions associated with ALS susceptibility. We performed interaction analysis by including cigarette smoking status (ever-smoker vs never-smoker) and a multiplicative SNP-smoking interaction term adjusting in covariate-adjusted models. Participants without smoking status were removed from this analysis. Interaction analyses could not be pursued using the validation data set because smoking status was unavailable.

### Transcriptome-Wide Association Analysis

We employed the widely used TWAS approach PrediXcan^29^ to predict the expression levels of participants from the Northern New England and Ohio ALS cohort. PrediXcan trains predictive models using reference data sets consisting of transcriptome and genotype information. Prediction weights were obtained from PredictDB, which derived these weights through the elastic net method using the Genotype-Tissue Expression version 7 as the reference panel.^29,30^ We examined associations between ALS risk and predicted gene expression levels across 15 tissues related to ALS, which included 13 brain and spinal cord regions (amygdala, anterior portion, caudate, cerebellar hemisphere, cerebellum, nucleus accumbens, cortex, frontal cortex BA9, hippocampus, hypothalamus, putamen, substantia nigra, C1 spinal cord), skeletal muscle, and whole blood tissues. We standardized predicted gene expression levels and tested associations using logistic regression with adjustment for sex, age at symptom onset, and the first 3 principal components of genetic ancestry. The false discovery rate (FDR) of 0.30 was used as the threshold for suggestively significant gene expression levels.

### Standard Protocol Approvals, Registrations, and Patient Consents

All participants involved were consented. All study procedures have been approved by the Committee for the Protection of Human Subjects at Dartmouth Health.

### Data Availability

We are in the process of uploading the genotype data used in this study to dbGaP with the accession number phs000101. Once processed, the data will be available through application on dbGaP.

## Results

### Genome-Wide Association Study Between ALS Risk and SNPs

**Figure 1:**
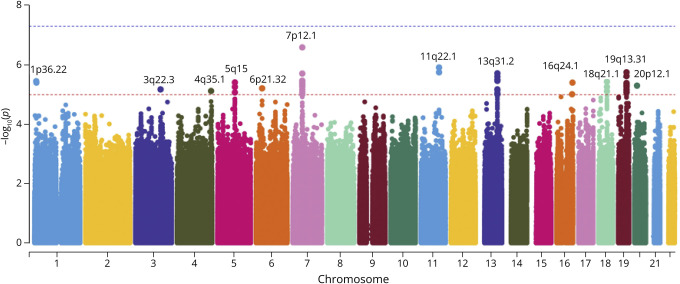
Manhattan Plot for SNPs in Northern New England and Ohio ALS Cohort The dashed line in blue indicates the significance threshold of 5E-8, and the line in red indicates the threshold of 1E-5. Twelve loci passed the suggestive significance threshold of 1E-5.

A total of 378 ALS cases and 235 controls of European ancestry passed the quality control. These participants' demographics information was provided in eTable 1. We calculated 14,125,267 association statistics for imputed genotypes. None of the SNPs passed the genome-wide significance threshold of 5E-8, but there were 150 SNPs with *p*-values less than 1E-5 (eTable 2). These 150 SNPs were localized to 12 cytogenetic locations (Figure 1).

The variants of suggestive statistical significance were all located within 15 genes. Table 1 lists the most statistically significant variant for each of the 15 genes. Among them, rs367543041, also known as c.1144G>A (p.A382T), is a missense variant of *TARDBP* (TAR DNA binding protein), which has been identified to be associated with ALS risk in multiple previous studies.^31-33^ This SNP was not available among imputed variants in the validation data set from dbGaP. Instead, we tested 2 nearby variants upstream and downstream, rs3835416 and rs148414479, as proxies. The association *p*-values for the 2 variants were 0.008 and 0.202, respectively.

**Table 1:** Fifteen Genes With Suggestive Significant SNPs in Northern New England and Ohio ALS Cohort

CHR	Gene	Cytogenetic band	SNP ID^a^	SNP position^b^	Alleles^c^	MAF^d^	Odds ratio	P^e^
1	*KIF1B*	1p36.22	rs12131785	1:10341516	C/T	0.19	0.48	3.57E-6
1	*TARDBP*	1p36.22	rs367543041	1:11082610	G/A	0.17	2.95	3.95E-6
3	*IL20RB*	3q22.3	rs79105994	3:136721632	A/T	0.44	0.56	6.67E-6
5	*MCTP1*	5q15	rs73133908	5:94085726	G/A	0.10	0.40	3.94E-6
6	*HLA-DMA*	6p21.32	rs129654	6:32916699	C/T	0.23	2.05	6.18E-6
11	*CNTN5*	11q22.1	rs7949592	11:99035893	G/A	0.12	0.40	1.23E-6
13	*LINC01047*	13q31.2	rs7490607	13:89868217	A/G	0.25	0.51	3.20E-6
13	*LINC00440*	13q31.2	rs12877053	13:89901248	T/C	0.25	0.50	1.94E-6
18	*MIR4527HG*	18q21.1	rs28505643	18:45081583	A/T	0.14	0.44	3.70E-6
19	*ZNF226*	19q13.31	rs35526214	19:44688732	TA/T	0.47	0.57	4.08E-6
19	*ZNF227*	19q13.31	rs2051059	19:44724723	T/C	0.45	0.57	4.41E-6
19	*ZNF233*	19q13.31	rs8106766	19:44769596	T/C	0.45	0.57	5.52E-6
19	*ZNF235*	19q13.31	rs2125579	19:44792701	G/T	0.45	0.57	5.25E-6
19	*ZNF112*	19q13.31	rs2722733	19:44845759	G/T	0.23	0.49	2.39E-6
20	*MACROD2*	20p12.1	rs67253970	20:14387190	G/C	0.23	2.07	5.03E-6

a This table lists the most significant SNP of each gene, and the other SNPs can be found in eTable 2.

b Positions are encoded in GRCh37/hg19.

c Major allele/minor allele (effect allele).

d Minor allele frequency (MAF).

e *p*- Values of SNPs were calculated from logistic regression adjusting for sex, age at symptom onset, and the first 10 principal components.

### Association Study Between ALS Risk and SNP-Smoking Interactions

**Figure 2:**
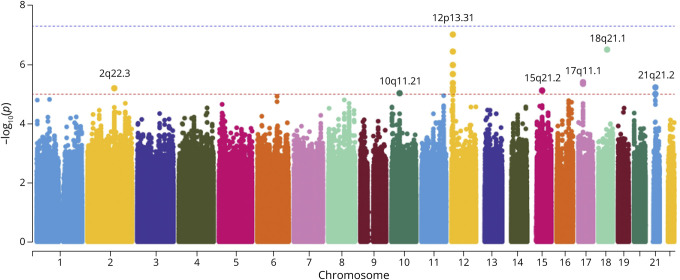
Manhattan Plot for SNP-Smoking Interactions in Northern New England and Ohio ALS Cohort The dashed line in blue indicates the significance threshold of 5E-8, and the line in red indicates the threshold of 1E-5. Seven loci passed the suggestive significance threshold of 1E-5.

For SNP-smoking interaction analysis, 276 ALS cases and 230 controls had available data on smoking status. We found 19 SNPs from 7 cytogenetic locations with evidence of interaction with smoking based on the P < 1E-5 threshold (Figure 2). None of the *p* values for interaction reached P < 5E-8.

Among these 19 SNPs, rs3815479 and rs201995562 are intronic variants located at the *GDF3* gene (growth differentiation factor 3) and *MYO5B* gene (myosin VB), as displayed in Table 2. The rest of the SNPs were not located within any genes. The entire list of the 19 SNPs with evidence of interaction with smoking is provided in eTable 3.

**Table 2:** Two Genes With Suggestive Smoking-Associated SNPs in Northern New England and Ohio ALS Cohort

CHR	Gene	Cytogenetic band	SNP ID	SNP position^a^	Alleles^b^	MAF^c^	Odds ratio	P^d^
12	*GFD3*	12p13.31	rs3815479	12:7843373	C/T	0.49	0.53	4.23E-6
18	*MYO5B*	18q21.1	rs201995562	18:47698658	C/A	0.33	2.19	3.13E-7

a Positions are encoded in GRCh37/hg19.

b Major allele/minor allele (effect allele).

c Minor allele frequency (MAF).

d *p*- Values of SNP-smoking interaction were calculated from logistic regression adjusting for sex, age at symptom onset, smoking main effect, and the first 10 principal components.

### Transcriptome-Wide Association Study of Tissue-Specific Predicted Gene Expression Levels and ALS Risk

We identified 8 genes from 5 tissues suggestively associated with ALS risk with FDR-adjusted P < 0.30, as displayed in Table 3. Higher predicted expression levels of *ZNF235* showed marginal significant associations with lower ALS risk in the brain caudate tissue (*p* = 6.76E-5, FDR = 0.14) and skeletal muscle tissue (*p* = 3.84E-5, FDR = 0.29). SNPs within *ZNF235* were also found to be associated with ALS risk in our GWAS analysis. The Miami plots, including both GWAS and TWAS results in brain caudate and skeletal muscle within the 1 Mb region of *ZNF235*, can be found in eFigure 2.

**Table 3:** Eight Gene Expressions Identified by TWAS to be Suggestively Associated With ALS Risk With FDR <0.3

Tissue	Gene	CHR	Cytogenetic band	Odds ratio	*p* Value	FDR^a^
Brain caudate	*CEP43* ^b^	6	6q27	0.68	8.10E-6	0.03
Brain caudate	*ZNF235*	19	19q13.31	0.71	6.76E-5	0.14
Brain hippocampus	*RPL7P18*	5	5q15	0.71	5.05E-5	0.14
Brain hippocampus	*ANO5*	11	11p14.3	0.72	1.35E-4	0.19
Brain hippocampus	*RP11-381K20.5*	5	5q31.2	1.38	2.23E-4	0.21
Brain substantia	*ZBTB14*	18	18p11.31	0.71	9.72E-5	0.20
Whole blood	*STIL*	1	1p33	0.64	3.36E-5	0.21
Skeletal muscle	*ZNF235*	19	19q13.31	0.70	3.84E-5	0.29

a FDR values were calculated in each tissue separately.

b The *CEP43* gene is also known as *FGFR1OP*.

## Discussion

In this study, we conducted GWAS and TWAS on ALS cases and controls from Northern New England and Ohio to explore the underlying genetic architecture of ALS. Our GWAS identified 15 genes associated with ALS risk, characterized by SNPs with suggestive significance (P < 1E-5), while TWAS identified 8 gene expression levels across 5 tissues suggestively associated with ALS risk (FDR <0.3).

For GWAS findings, we identified a suggestively significant variant, rs367543041, located within the ALS-associated gene *TARDBP*. This result aligns with previous studies reporting rs367543041 as an ALS risk variant.^31-33^ The *TARDBP* variation, particularly the p.A382T missense variant (rs367543041), has been linked to approximately 30% of ALS cases within the genetically conserved Sardinian population.^32,33^ Although we could not directly replicate this variant in the larger dbGaP data set because this SNP was not included in the imputed genotype, we observed an association with a nearby proxy SNP, supporting the involvement of *TARDBP* variation in ALS susceptibility. The GWAS also identified *KIF1B*, a member of the kinesin family, associated with ALS risk.^34^ While there is no direct evidence linking *KIF1B* variants to ALS,^35^ a study observed a differential regulation of *KIF1B* in sciatic nerve cells and the spinal cord, suggesting its potential significance in ALS.^36^ In addition, another member of the kinesin family, *KIF5A*, has been identified to be associated with ALS in previous studies.^10,37^ Another gene identified as suggestively significant in the GWAS is *MACROD2*, a mono-ADP ribosylhydrolase that responds to DNA damage by nuclear export to the cytoplasm.^38^ In previous studies, *MACROD2* has been reported as a neurodevelopmental-related gene,^39,40^ recognized as a susceptibility gene for autism spectrum disorders and schizophrenia.^39,41^

We additionally explored SNP-smoking interactions, identifying variants in *GDF3* and *MYO5B* as suggestively interacting with cigarette smoking to influence ALS risk. *GDF3* is a member of growth differentiation factors, which constitutes a subfamily of the transforming growth factor-β (TGF-β) superfamily.^42,43^ *GDF3* has been identified as an inhibitor of bone morphogenetic proteins (BMPs).^44^ BMPs play a key role in inducing the formation of cartilage, bone, and skeletal muscle.^45,46^ The participation of *GDF3* in the development of bone and cartilage, acting as an inhibitor of BMPs, may provide insights into its potential association with ALS. *MYO5B* is a member of the class V myosins participating in intracellular transport.^47,48^ Another member of the class V myosins, *MYO5C*, showed an association with late-onset AD based on its gene expression level.^49^ Despite SNP-smoking interactions being identified for *GDF3* and *MYO5B*, our validation cohort's lack of smoking data precluded replicating these findings. In future analyses, we plan to leverage geographic information system technology and pollution databases from government agencies to explore more potential gene-environment interactions within our cohort, including lead, mercury, pesticides, and air pollution.

For TWAS findings, our results indicate *ZNF235* as a potential ALS risk gene. *ZNF235* encodes a zinc finger protein that acts as a transcriptional repressor, potentially participating in neuronal differentiation.^50,51^ Both GWAS and TWAS analyses suggest associations between *ZNF235* and ALS, with predicted *ZNF235* expression levels in the caudate and skeletal muscle tissues showing reduced expression levels among ALS cases. Despite limited knowledge regarding its role in ALS, our results suggest *ZNF235* as a candidate gene warranting further functional investigation. Another TWAS-identified gene, *CEP43* (also known as *FGFR1OP*), is a fusion partner for *FGFR1*.^52^ A previous study indicates that *FGFR1* can mediate motor neuron apoptosis in ALS.^53^

Our study had several limitations. The modest sample size likely constrained our power to detect genome-wide significant associations. As a result, our study failed to identify significant associations with other known ALS genetic risk factors, such as *SOD1*, *NUP50*, and *ERBB4*, possibly because of differences in population structure or sample size. To mitigate this limitation, we sought to supplement our cohort with a larger dbGaP data set, but this introduced challenges with genotyping platform differences and unavailable smoking data. Future studies with expanded sample sizes and harmonized genotyping arrays are needed. This study was restricted to individuals of European ancestry. Future research should also include more populations to validate or extend our findings across different ancestral groups and geographical regions. In addition, the significant variant in *TARDBP* was not directly validated in the dbGaP database, so we can only conclude it as a tentative association. Despite these constraints, our integrated GWAS and TWAS of ALS provided useful insights into genetic susceptibility. Moreover, TWAS enabled us to explore the gene expression levels across 15 potential ALS-related tissues, including 13 brain-related tissues, skeletal muscle tissue, and whole blood tissue, to understand better the underlying genetic mechanisms of ALS in different tissues.

In summary, this study identified variants and genes associated with ALS risk through GWAS and TWAS analyses. We validated the *TARDBP* association and identified *ZNF235* as a potential novel ALS risk gene. Our findings also reinforce the likely complex interplay of genetic and environmental factors in ALS etiology. Follow-up genetic research is important to uncover how identified variants and genes influence motor neuron degeneration in ALS.

## Appendix Authors

**Table:**

Name	Location	Contribution
Siting Li, PhD	Departments of Biomedical Data Science and Epidemiology, Dartmouth College, Hanover, NH	Drafting/revision of the manuscript for content, including medical writing for content; study concept or design; analysis or interpretation of data
Jiang Gui, PhD	Department of Biomedical Data Science, Dartmouth College, Hanover, NH	Drafting/revision of the manuscript for content, including medical writing for content; study concept or design; analysis or interpretation of data
Michael N. Passarelli, PhD	Department of Epidemiology, Dartmouth College, Hanover, NH	Drafting/revision of the manuscript for content, including medical writing for content; study concept or design
Angeline S. Andrew, PhD	Dartmouth Health, Lebanon, NH	Drafting/revision of the manuscript for content, including medical writing for content; study concept or design; analysis or interpretation of data
Kathleen M. Sullivan, MBA	Dartmouth Health, Lebanon, NH	Drafting/revision of the manuscript for content, including medical writing for content; major role in the acquisition of data
Kevin A. Cornell, MS	Dartmouth Health, Lebanon, NH	Drafting/revision of the manuscript for content, including medical writing for content; major role in the acquisition of data
Bryan J. Traynor, MD, PhD	Neuromuscular Diseases Research Section, National Institute on Aging; National Institute of Neurological Disorders and Stroke, National Institutes of Health, Bethesda; RNA Therapeutics Laboratory, National Center for Advancing Translational Sciences, National Institutes of Health, Rockville, MD	Drafting/revision of the manuscript for content, including medical writing for content; major role in the acquisition of data; study concept or design; analysis or interpretation of data
Ali Stark, BS	Neuromuscular Diseases Research Section, National Institute on Aging, National Institutes of Health, Bethesda, MD	Drafting/revision of the manuscript for content, including medical writing for content; major role in the acquisition of data
Ruth Chia, PhD	Neuromuscular Diseases Research Section, National Institute on Aging, National Institutes of Health, Bethesda, MD	Drafting/revision of the manuscript for content, including medical writing for content
Rebecca M. Kuenzler, MD	Cleveland Clinic, OH	Drafting/revision of the manuscript for content, including medical writing for content
Erik P. Pioro, MD, PhD	Department of Medicine, University of British Columbia, Vancouver, BC, Canada	Drafting/revision of the manuscript for content, including medical writing for content
Walter G. Bradley, DM, FRCP	University of Miami Miller School of Medicine, Miami, FL	Drafting/revision of the manuscript for content, including medical writing for content; study concept or design
Elijah W. Stommel, MD, PhD	Dartmouth Health, Lebanon, NH; Department of Neurology, Geisel School of Medicine at Dartmouth, Hanover, NH	Drafting/revision of the manuscript for content, including medical writing for content; major role in the acquisition of data; study concept or design

## Glossary

AD: Alzheimer disease
ALS: amyotrophic lateral sclerosis
BMP: bone morphogenetic protein
FDR: false discovery rate
FTD: frontotemporal dementia
GWAS: genome-wide association study
PD: Parkinson disease
SNP: single nucleotide polymorphism
TWAS: transcriptome-wide association study
